# Prioritising health service innovation investments using public preferences: a discrete choice experiment

**DOI:** 10.1186/1472-6963-14-360

**Published:** 2014-08-28

**Authors:** Seda Erdem, Carl Thompson

**Affiliations:** Economics Division, Stirling Management School, University of Stirling, Cottrell Building, Stirling, FK9 4LA UK; Department of Health Sciences, University of York, SRB, Heslington, York YO10 5DD UK

## Abstract

**Background:**

Prioritising scarce resources for investment in innovation by publically funded health systems is unavoidable. Many healthcare systems wish to foster transparency and accountability in the decisions they make by incorporating the public in decision-making processes. This paper presents a unique conceptual approach exploring the public’s preferences for health service innovations by viewing healthcare innovations as ‘bundles’ of characteristics. This decompositional approach allows policy-makers to compare numerous competing health service innovations without repeatedly administering surveys for specific innovation choices.

**Methods:**

A Discrete Choice Experiment (DCE) was used to elicit preferences. Individuals chose from presented innovation options that they believe the UK National Health Service (NHS) should invest the most in. Innovations differed according to: (i) target population; (ii) target age; (iii) implementation time; (iv) uncertainty associated with their likely effects; (v) potential health benefits; and, (vi) cost to a taxpayer. This approach fosters multidimensional decision-making, rather than imposing a single decision criterion (e.g., cost, target age) in prioritisation. Choice data was then analysed using scale-adjusted Latent Class models to investigate variability in preferences and scale and valuations amongst respondents.

**Results:**

Three latent classes with considerable heterogeneity in the preferences were present. Each latent class is composed of two consumer subgroups varying in the level of certainty in their choices. All groups preferred scientifically proven innovations, those with potential health benefits that cost less. There were, however, some important differences in their preferences for innovation investment choices: Class-1 (54%) prefers innovations benefitting adults and young people and does not prefer innovations targeting people with ‘drug addiction’ and ‘obesity’. Class- 2 (34%) prefers innovations targeting ‘cancer’ patients only and has negative preferences for innovations targeting elderly, and Class-3 (12%) prefers spending on elderly and cancer patients the most.

**Conclusions:**

DCE can help policy-makers incorporate public preferences for health service innovation investment choices into decision making. The findings provide useful information on the public’s valuation and acceptability of potential health service innovations. Such information can be used to guide innovation prioritisation decisions by comparing competing innovation options. The approach in this paper makes, these often implicit and opaque decisions, more transparent and explicit.

## Background

Innovations – products, practices, methods or services that are perceived as “new” by adopters and potential users [1] – come in various forms. In systems where the number of innovations that can be implemented outstrips scarce resources, prioritisation surrounding which health service innovations receive resources is inevitable [2, 3]. Alongside the use of economic criteria (e.g., cost-effectiveness, cost-utility), other factors, such as ease of implementation, severity of disease, and political acceptability [4] are also used in the prioritisation of health service innovations, often implicitly. Recently, health systems have sought to incorporate public preferences in priority setting and investment decisions [5–9].

Studies examining the public’s priorities for spending on healthcare often focus on criteria such as ‘severity of illness’ [10, 11] ‘age’ [12], and ‘value for money’ [10]. Often these criteria are viewed in isolation from each other. In this paper, we start from a descriptive position that innovation investment prioritisation decisions are complex and a normative position that they should be based on multiple criteria; in doing so, we propose a means of identifying the importance attached to such criteria. We use the term ‘characteristic’ to represent such criteria. Every innovation has several such characteristics: how much it costs, the health benefits likely to result from implementation, the population targeted or most affected. In this paper, we explore the acceptability of (and importance attached to) health service innovations and their characteristics from a social perspective. We aim to explain how valuation of innovation characteristics can be meaningfully used as a guide for prioritisation and innovation investment. Crucially, we want to fill one of the important gaps in the innovation implementation literature: how the public feel about potential choices made on their behalf.

Our methodological approach, Discrete Choice Experiments (DCE), involves viewing healthcare innovations as ‘bundles’ of characteristics (e.g., their cost, how long they take to implement, and their likely impact on health), which allows us to study a wide range of innovations sharing the same characteristics (e.g., cost), but at different levels (e.g., £10, £20), without specifying exactly what these innovations are. By using this broader framework, we aim to help policy-makers and other decision-makers in prioritising the innovation investment choices available to them. Additionally, whilst DCE is receiving growing attention in health economics [13–16], its use in an implementation (of innovations) context is sparse [10, 11, 13]. To the best of our knowledge, this is the first empirical study to explore public preferences for health service innovation investment options.

## Methods

### Discrete choice experiments (DCE)

The Discrete Choice Experiment (DCE) is a technique for eliciting individuals’ preferences. It is commonly used in environmental economics [17–19] and transportation [20–22] for prioritisation decisions using public preferences. Health economists have begun to see the benefit of the approach for helping answer a wide range of policy questions [13, 23], including priority setting frameworks [2, 10, 24–27].

DCE involves asking respondents to choose between competing (hypothetical) innovations, presented as scenarios, using a series of defined characteristics (or attributes) represented at various levels (e.g., an attribute of ‘financial cost’ at the level of ‘£10’). Using the choice data, the relative importance of specific, plausible scenarios, and the values individuals attach to their constituent parts (i.e., willingness-to-pay) are estimated via probabilistic choice models [28, 29].

### Attribute and level selection

Attributes and their associated levels were identified from literature reviews and policy documents (e.g., NICE [30]), interviews with Foundation Trust managers and Trust members, and a focus group discussion with non-academic staff in York^a^. Innovation characteristics from these sources were compiled and refined using interviews with managers from a large NHS Foundation Trust. The final selection of characteristics to be used in the survey is based on the focus group discussions and discussions with managers from a NHS Foundation Trust. As a result, we used six health service innovation attributes in the surveys: (i) target population; (ii) age group; (iii) time to get into practice; (iv) whether it works; (v) potential health benefits; and, (vi) cost to an individual taxpayer. The detailed descriptions are presented in Table 1. The survey attributes, the number of choice tasks, and survey question framing were further tested using two pilot surveys^b^. The pilot surveys yielded data that could be used to identify confusions, lack of understanding, and the time needed to complete the survey. They also tested the framing of the information, which is provided to respondents before they took the survey, about innovations, innovation characteristics and levels. The study was approved by the Health Sciences Ethics and Governance Committee of the University of York.

**Table 1:** **Health service innovation attributes and levels**

Attribute	Levels
Target population	People with disability
	People with cancer
	People with mental health problems
	People with obesity
	People with asthma
	People with drug addictions
Age group	Young (less than 18)
	Adults (18–65)
	Elderly (more than 65)
Time to get into practice	0–5 months
	6–12 months
	More than 12 months
Whether it works	It works and scientific studies confirm this
	It works but not scientifically proven
	Experts say it works elsewhere in the NHS
Potential health benefit/gain	Best health (100%)
	Good health (75%)
	Moderate health (50%)
Cost to you as a taxpayer (£/month)	£10, £20, £30, and £40

Various reasons lay behind the final selection of these attributes. Different health issues differentially impact on certain population and age groups. Thus, expressed need with potential for improved health between groups may also be different [31]; as will their capacity to benefit [30], p.47]. Age is a contentious decision criterion for health service prioritisation [32–34]. We included ‘age’ to investigate whether, as is often assumed rather than established, there are systematic differences in the public’s preferences for innovations targeting certain age groups.

Time to get the innovation adopted into practice differs between units of adoption (e.g., small teams or large organisations) [35]. It might also depend on innovation type; some innovations may be more complex, requiring a longer time to be implemented, others may require additional time for training. However, the quicker an innovation is adopted the sooner the public will be exposed to its assumed benefits. Thus, we wanted to explore whether the public would be willing to trade off potential health benefits in three time-periods – described as realistic by an NHS management team, (0–5 months, 6–12 months, and more than a year).

The strength of the evidence underpinning effectiveness is a determinant of innovation diffusion and adoption [36, 37]. We described the evidence on innovation effectiveness using an approach akin to Farrar *et al.* [38] and Cunningham *et al.* [39]: three levels, with differing levels of certainty and strength (inferred from an accompanying information source).

**Figure 1:**
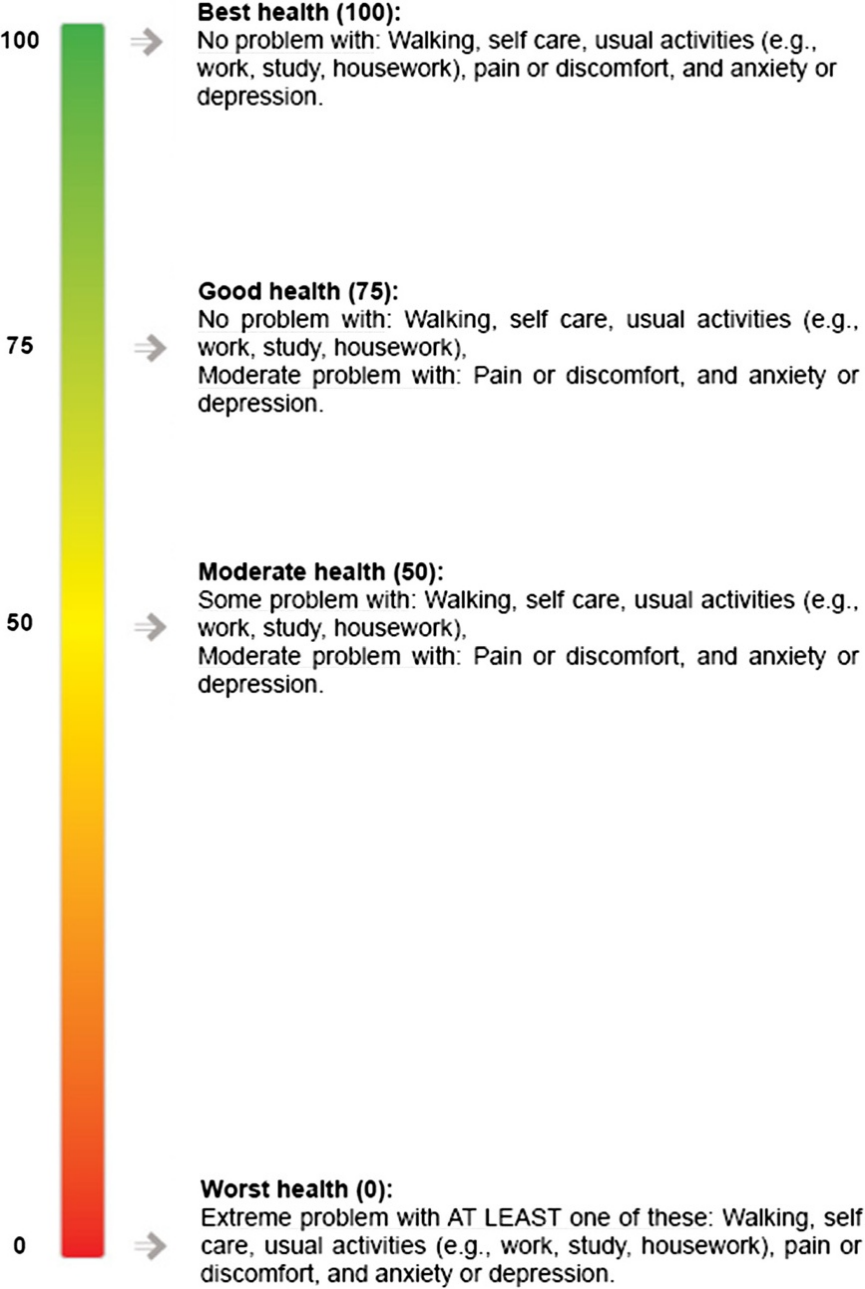
**Visual health status scale.**

The potential health gain generated by an innovation is also an important factor for decision-makers when prioritising innovations for implementation [10, 40, 41]. There is strong evidence that health gain also matters to the public judging the allocation of healthcare resources [34, 42, 43]. However, measuring health gain is challenging as information on those that benefit most from an innovation, and how much benefit will accrue, is often absent. Green and Gerard [10] overcome this limitation by using qualitative categories of changes in health (e.g., small, medium and large gains); an approach that leaves open the possibility that people will interpret categories differently. Quantitative approaches (e.g., Quality Adjusted Life Years gained or number of patients treated) have also been used in priority setting [40, 44]. However, the lack of reliable data, difficulty in generalising to future applications, and explaining quantitative measures to a society which some commentators claim is functionally ‘innumerate’ [45] remain considerable barriers to popular use. We quantified potential change in health status using a multi-attribute health status classification system, EQ-5D [46] and presented it using a visual ‘health status scale’, as shown in Figure 1.

EQ-5D has five dimensions; mobility, self-care, usual activities, pain/discomfort, and anxiety/depression. Each dimension has three levels; no problem, some problems, and extreme problems, resulting in 243 ‘scored’ health states. For example, perfect health (no problems on any dimensions) would result in a score of 100 (20 in each dimension). Conversely, death (more detail on different states and scores can be found in EuroQol [46]) would be represented by a score of 0. We needed to select a feasible number of heath states (attributes) from the 243 possibilities. These needed to be realistic, clearly understood by respondents and easily discriminated. Small differences in scores were unlikely to meet these criteria and so we selected four health states – worst health (score 0), moderate health (score 50), good health (score 75), and best health (score 100), labelled ‘common core’ health states (EuroQol [46], p.31). Our pilot surveys showed these four health states were easy to understand and differentiate.

Office of Health Economics (OHE) health care expenditure data provided the basis for the cost attribute. Annual healthcare expenditure per person in 2012 was calculated at circa £2400. Extra money for innovation implementation was assumed to be increments of the health care expenditure per person per month: 5% (£10), 10% (£20), 15% (£30), and 20% (£40). The survey implied that this would be an on-going payment from their tax payments^c^. Pilot surveys and interviews with health professionals confirmed these levels as appropriate.

### Experimental design

The experimental design was created using *Ngene* [47]. It involved obtaining priors from pilot surveys^b^. For the pilot surveys, a pivot design, minimising D-error (a measure of efficiency) was generated using priors of zero for the marginal utility of all attributes. Choice models estimated from the pilot data provided new estimates of the marginal utilities. These point estimates and their standard errors were used as priors in a new Bayesian efficient design [48] for the main survey. The pilot surveys ensured the validity of the DCE design before the final administration.

**Figure 2:**
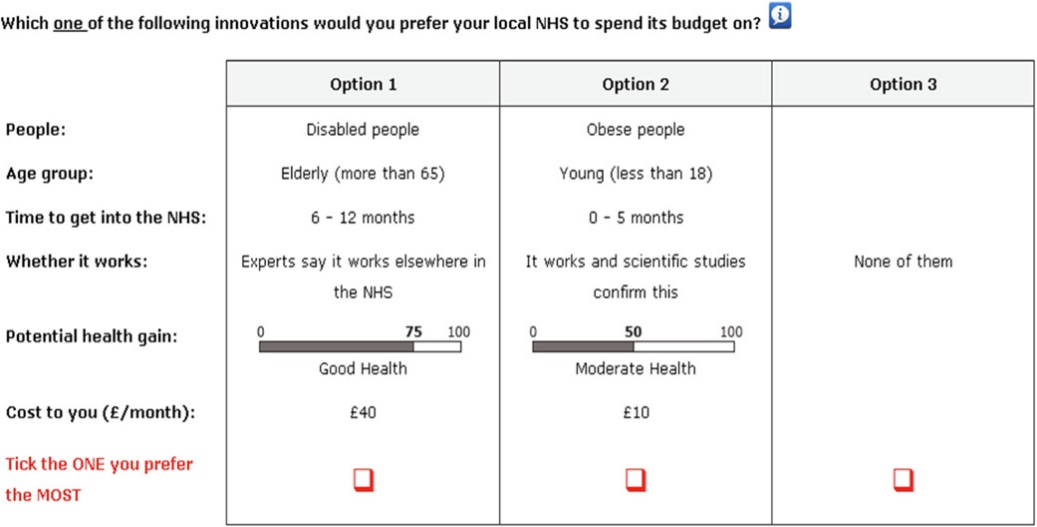
**An example DCE task.**

The final design consisted of five blocks, each having 12 choice tasks. Each respondent was randomly assigned to a block. For each choice task, respondents were asked to choose between two hypothetical innovation scenarios that they thought the NHS should prioritise and a ‘none of them’ option. The ‘none of them’ option is included to reflect real choices and the consistency with consumer theory. An example choice task is presented in Figure 2.

### Data collection

We conducted postal questionnaires with the general public in West Yorkshire, UK, in 2011. For model estimation we used 3,000 observations gathered from a sample of 250 respondents^d^, each responded 12 choice tasks. The sample was randomly selected from the Electoral Register data and a single NHS Foundation Trust database. We used stratified random sampling from postcodes for which we had indices of deprivation and ethnic density.

Most respondents were female (61%), with a mean age of 50 years, and the majority of them described themselves as ‘white’ (87%). Approximately, 30% were retired and 28% were employed full-time. The 2011 UK census data suggests that people in our sample were similar to the West Yorkshire population but a little older than the regional average (mean, 40 years) and more likely to be retired (West Yorkshire average of 15%).

### Model

We used scale-adjusted Latent Class (LC) models to provide in-depth information on how public preferences and values attached to innovation options vary with individual characteristics.

The underlying theory of the LC models posits that individuals’ choice behaviour and preferences can be allocated into a set of *Q* latent classes. Preferences within each class are assumed to be homogenous, but allowed to differ across classes. While traditional LC models potentially confound heterogeneity in consumer preferences and individual differences in error variance [49–51], scale-adjusted LC choice models separately consider both types of response heterogeneity by accommodating the possibility that each latent class may be composed of subsets of respondents who differ in terms of their level of variance [52–54]. If this confounding issue is not handled in the modelling, the predictions obtained may then contain additional amount of error as well as potential bias [53].

In scale-adjusted LC model it is assumed that within each latent class there are s classes with different values of scales. The scale parameter can be explained in terms of the variance in observed responses. Response variance within each s class is related to the scale parameter: *σ*_*s*_^2^ = *π*^2^/6*λ*_*s*_^2^. This can be interpreted as a measure of ‘uncertainty’ or ‘lack of certainty’ [52–54]. For respondents with the scale approaching zero means that the response variance approaches to infinity, which is considered as complete uncertainty [53]. The higher the scale gets, the higher the level of certainty is. Accounting for the scale heterogeneity within each class, the probability of option *i* among *J* alternatives, chosen by respondent *n* belonging to class *q*, can be expressed in MNL form as the following:
1

where *π*_*s*_ are the scale membership probabilities within class *q* and *λ*_1_ is normalised to 1 for identification purposes; *β*_*q*_ is a class-specific vector of coefficients for *X*_*nit*_ characteristics of innovations in choice set *t*_*n*_ = 1, …, *T*.

As individuals make a series of choices, the contribution of individual *i* to the likelihood is the joint probability of the sequence y_*n*_ = [y_*n*1_, …, y_*nT*_], becomes the following:
2

The class assignment of the individuals is latent, and is thus not known to the analyst. However, following Swait [55] and Boxall and Adamowicz [56], a latent membership likelihood function classifies individuals into one of the *Q* segments, with a probability of *π*_*n*|*q*_. The classification variables used in this function can relate to individuals’ characteristics, such as gender^e^. The choice model for individual *n* is then the expectations of the class specific contributions, which is computed by taking the product of the joint probability, *P*_*yn*|*q*_, and the probability of class membership, *π*_*n*|*q*_:
3

This model allows us to explain individuals’ choice behaviour from their choice attributes and simultaneously show how their characteristics, such as gender influence class membership. We then maximize the log-likelihood function,  with respect to the parameters to be estimated (i.e., *β*_*q*_ and latent class parameters) via Maximum Likelihood estimation, where *N* is the number of individuals. The analysis was performed using Latent GOLD Syntax version 4.5.

As there is no one superior criterion on choosing the optimum number of latent classes, we considered a number of factors in deciding the class number. We estimated models with different classes and used improvements in values of information criteria –Bayesian Information Criterion (BIC) and Akaike Information Criterion (AIC) and log-likelihood values [56, 57], and model parsimony as statistical guidance when comparing the models. Generally, when the number of classes increases, model fit gets better, the log-likelihood values increase, and the information criteria values decrease. When an additional segment is added to the model, the model fit is penalised for the increase in the number of parameters due to the additional segment. The model with additional segment that is beyond the optimal one may not provide much improvement.

The public’s valuation of innovation characteristics, i.e., marginal willingness to pay (WTP), is then given by the (negative) ratio of the attribute parameter to the parameter on the cost of innovations. Because the impact of the attribute is not predetermined, WTP can be either positive or negative. Negative WTP becomes the amount they are willing to accept in compensation to suffer a utility reducing attribute change. For ease of interpretation we will use the abbreviation WTP, with the sign of the effect indicating the nature of the impact of the attribute.

## Results

The results from the analysis of the choice data is presented in Table 2. As a point of reference our analysis starts with the standard Multinomial Logit (MNL) model that assumes homogeneity of preferences and error variances in our sample. We then analyse preference heterogeneity using a scale-adjusted Latent Class (LC) model, in which we identify three latent classes and two scale factor levels. A three-class parsimonious model with two scale levels is preferred for two reasons. First, adding an additional segment and a scale level beyond the preferred levels did not seem to provide much improvement in the values of information criteria. Second, when a four-class model is used, more than half of the parameters in the additional class are not statistically significant, indicating that these parameters do not seem to have significant effect on consumers’ choices.

**Table 2:** **Analysis results**

	MNL		Scale-adjusted latent class					
Parameters			Class-1 (54%)		Class-2 (34%)		Class-3 (12%)	
	coef.	s.e.	coef	s.e.	coef	s.e.	coef	s.e.
ASC	−1.270*	0.076	−1.172*	0.133	−2.146*	0.208	0.549**	0.247
Cost	−0.031*	0.002	−0.036*	0.003	−0.036*	0.003	−0.036*	0.003
**Target people:**								
People with disability	0.590*	0.065	0.832*	0.146	0.086	0.084	0.479**	0.225
People with drug addiction	−1.570*	0.083	−2.232*	0.231	−0.442*	0.135	−1.775**	0.715
People with cancer	1.560*	0.119	2.017*	0.277	0.645*	0.171	1.706*	0.371
People with mental health problems	0.310*	1.728	0.677*	0.154	−0.070	0.134	−0.622**	0.271
People with obesity	−1.150*	0.078	−1.583*	0.161	−0.450*	0.107	0.094	0.276
People with asthma	0.260*	0.085	0.289**	0.120	0.231***	0.138	0.118	0.256
**Target age:**								
Young (less than 18)	0.090**	0.040	0.175*	0.054	0.036	0.046	−0.422**	0.212
Adults (18–65)	0.180*	0.041	0.274*	0.068	0.071	0.051	−0.175	0.166
Elderly (more than 65)	−0.270*	4.300	−0.449*	0.074	−0.107**	0.052	0.597*	0.163
**Implementation time:**								
Between 6–12 months	−0.027	0.038	0.027	0.046	−0.075***	0.042	0.164	0.132
More than 12 months	−0.029	0.038	−0.036	0.046	0.015	0.040	−0.254	0.163
Between 0–5 months	0.056	1.604	0.009	0.045	0.060	0.044	0.090	0.123
**Whether it works:**								
It works but not scientifically proven	−0.250*	0.041	−0.201*	0.060	−0.194*	0.052	−0.417**	0.164
It works and scientific studies confirm this	0.210	5.612	0.175*	0.046	0.113**	0.045	0.295**	0.125
Experts say it works elsewhere in the NHS	0.040	0.039	0.026	0.051	0.081***	0.044	0.122	0.131
**Potential health benefits:**								
Moderate health (50%)	−0.360	−8.731	−0.283*	0.060	−0.261*	0.056	0.015	0.130
Good health (75%)	0.110*	0.037	0.061	0.041	0.072**	0.037	−0.011	0.122
Best health (100%)	0.250*	0.038	0.222*	0.061	0.189*	0.050	−0.004	0.121
**Scale**			λ_1_ = 1, λ_2_ = 2.65 (s.e. = 0.375)					
**Probabilities**								
Class size			0.541*	0.040	0.338	0.037*	0.121*	0.026
Scale λ_1_			0.652* (s.e. = 0.086)					
Log-likelihood	−2704.018		−2383.155					
Pseudo R-squared	0.20		0.39					
Number of parameters	15		47					
Number of observation	3000		3000					

Variables effects-coded. For clarity, we also present the baseline levels, which are arbitrarily chosen. *significant at p < 0.01; **significant at p < 0.05, ***significant at p < 0.10.

Results of the standard MNL model show that respondents prefer the implementation of innovation options to a ‘no-innovation’ option. They prefer innovations that are scientifically proven, targeting young and adults (between 18 and 65), that result in health gains and cost less. They do, however, vary in their preferences for innovations aimed at specific population groups: preferring investment in innovations for people with ‘disability’ , ‘cancer’ and ‘asthma’ , but not preferring investment in innovations targeting people with ‘drug addiction’ and ‘obesity’.

Moving from the MNL model to the scale-adjusted LC model shows substantial improvement in the model fit (around 321 log-likelihood units). This suggests that there are some people showing different preferences with different error variances (or “choice uncertainty”). All else being equal, Class-1 accounts for the majority of the sample (54%), Class-2 accounts for 34%, and Class-3 accounts for 12% of the sample. Each latent class is composed of two consumer subgroups, the first (*λ*_1_ = 1) expressing a higher variance than the other (*λ*_2_ = 2.65) and accounts for the majority of the respondents in each class (65%). Notwithstanding that the level of “certainty” can be a function of various things, following the interpretation in Magidson and Vermunt [53], Flynn et al. [52], and Campbell et al. [54], we can say that 35% of the respondents have a higher level of certainty in their choices. Additionally, socio-economic variables, such as gender, employment status, education used in the class membership indicate that the classes are made up of different profiles of respondents. However, we find no differences in the class memberships according to respondents’ demographics; thus, we exclude them from the analysis.

Similar to the MNL results, people in all three classes prefer innovations that are scientifically proven, have potential health benefits and cost less. However, there are some important differences. Although the alternative specific constants (ASC) for the ‘none’ option are negative and significant for Class-1 and 2 (88% respondents) - implying these respondents, all else being equal, prefer the implementation of innovation options - a minority, (12%) prefers “no-innovation” option. This variation across the three classes is also reflected in their preferences for innovations targeting different population groups. Whilst all classes feel the NHS should invest in innovations targeting people with ‘cancer’ and do not prefer funding innovations targeting people with ‘drug addictions’, people differ in their preferences for innovations that target those with ‘disability’ , ‘mental health problems’, ‘obesity’ and ‘asthma’. Class-1 and Class-3 are significantly and positively predisposed towards innovations targeting people with ‘disability’ , Class-2, however, has no significant preferences for innovations targeting these people. While Class-1 prefers innovations targeting people with ‘mental health problems’ , Class-2 has indifferent preferences and Class-3 has negative preferences for such innovations. Class-3 particularly has no significantly different preferences for spending on innovations targeting people with ‘obesity’; Class-1 and Class-2, however, are significantly and negatively orientated towards innovations targeting obesity. As for the innovations targeting people with ‘asthma’ , while Class-1 shows significant and positive preferences for such innovations, Class-2 and Class-3 show no significant preferences for innovations targeting these people.

Preferences for innovations targeting different age groups also vary at each class. While Class-1 prefers innovations targeting adults the most, followed by young people relative to elderly, Class-2 and Class-3 hold different preferences: Class-2 does not prefer innovations targeting elderly and is indifferent between other age groups, Class-3 prefers spending on elderly, but not on young people.

The results also showed that the respondents do not differ on their valuation of the speed of implementation; they are all indifferent between different implementation times. When it comes to uncertainty surrounding the likely effect of an innovation, all respondents value innovations back by scientific studies (with significant negative preferences for innovations not scientifically proven). Only Class-2 shows significant positive preferences towards expert opinions.

As for the potential health benefits of innovations, all else being equal, the majority of the sample (i.e., Class1 and Class-2) prefer improvement from a worst health to the good health (75%), but do not prefer having a moderate health (50%) gain. Only Class-3, 16% of the sample, is indifferent in the levels of health benefits. To further develop our understanding of respondents’ preferences we calculate their valuations of innovation characteristics (i.e., WTPs), which allows for comparisons of their preferences within and between classes.

### Willingness–to-pay (WTP) estimates

Table 3 compares the marginal WTP results derived under the two models, using the ratios  As can be seen, the public’s valuations of innovation characteristics are aligned with their preferences. Irrespective of the model assumption shown in Table 2, respondents are willing to pay most for innovations that are scientifically proven, have at least moderate health benefit, take less time to implement, and target adults and the young. Comparing the marginal WTP estimates obtained from the MNL and the weighted average of scale-adjusted LC model, with respect to their baselines, reveal some significant differences between the respondents’ valuations of innovations targeting people with obesity [p-value = 0.026] and innovations resulting in ‘good’ [p-value =0.007] and ‘best possible’ health gains [p-value =0.005].

**Table 3:** **Marginal WTP estimates (£/month)**

	MNL	Class-1 (54%)	Class-2 (34%)	Class-3 (12%)	Weighted average
**Target people:**	9.31 (1.84 – 16.79)	4.28 (−6.53 – 15.09)	4.31 (−5.22 – 13.85)	30.41 (10.86 – 49.96)	7.46 (0.61 – 14.31)
People with disability					
People with drug addiction	−61.01 (−69.38 – -52.64)	−80.38 (−96.12 – -64.64)	−10.25 (−21.97 – 1.46)	−31.86 (−76.58 – 12.87)	−50.82 (−60.60 – -41.03)
People with cancer	40.77 (32.06 – 49.48)	37.00 (18.49 – 55.51)	19.77 (8.20 – 31.34)	64.29 (38.51 – 90.07)	34.49 (23.72 – 45.26)
People with mental health problems	Baseline level				
People with obesity	−47.28 (−55.43 – -39.13)	−62.43 (−73.46 – -51.40)	−10.49 (−20.92 – -0.07)	19.76 (−1.90 – 41.42)	−34.93 (−42.00 – -27.85)
People with asthma	−1.51 (−9.83 – 6.81)	−10.74 (−20.04 – -1.44)	8.32 (−3.49 – 20.13)	20.42 (0.58 – 40.26)	−0.53 (−7.19 – 6.14)
**Target age:**					
Young (less than 18)	11.61 (7.16 – 16.06)	17.22 (11.24 – 23.20)	3.92 (−0.61 – 8.45)	−28.11 (−46.48 – -9.74)	7.23 (3.27 – 11.19)
Adults (18–65)	14.41 (9.82 – 18.99)	19.94 (12.82 – 27.07)	4.90 (−0.10 – 9.89)	−21.30 (−34.94 – -7.65)	9.86 (5.57 – 14.15)
Elderly (more than 65)	Baseline level				
**Implementation time:**					
Between 6–12 months	−2.69 (−6.81 – 1.44)	0.48 (−3.71 – 4.68)	−3.76 (−7.92 – 0.40)	2.05 (−8.67 – 12.77)	−0.76 (−3.61 – 2.09)
More than 12 months	−2.76 (−6.88 – 1.37)	−1.24 (−5.43 – 2.96)	−1.28 (−5.10 – 2.55)	−9.51 (−23.37 – 4.36)	−2.25 (−5.18 – 0.67)
Between 0–5 months	Baseline level				
**Whether it works:**					
It works but not scientifically proven	−14.66 (−18.88 – -10.44)	−10.41 (−15.52 – -5.30)	−8.49 (−13.18 – -3.80)	−19.68 (−33.83 – -5.53)	−10.89 (−14.63 – -7.14)
Experts say it works elsewhere in the NHS	−1.65 (−16.82 – 13.51)	−4.14 (−8.20 – -0.09)	−0.91 (−4.77 – 2.96)	−4.79 (−15.39 – 5.81)	−3.13 (−5.85 – -0.41)
It works and scientific studies confirm this	Baseline level				
**Potential health benefits:**					
Moderate health (50%)	Baseline level				
Good health (75%)	15.45 (11.09 – 19.82)	9.51 (5.02 – 14.01)	9.20 (4.83 – 13.56)	−0.71 (−12.31 – 10.89)	8.17 (5.10 – 11.24)
Best health (100%)	20.11 (15.61 – 24.61)	13.94 (7.79 – 20.09)	12.44 (7.08 – 17.80)	−0.53 (−12.90 – 11.84)	11.68 (7.87 – 15.49)

Figures in parentheses are confidence intervals at the 95% level, obtained using the delta method. Baseline levels are chosen arbitrarily.

The marginal WTP of respondents in the three classes also show some variations. Relative to the people with ‘mental health problems’, all classes are, on average, willing to pay for innovations targeting cancer patients. While Class-3, on average, shows the highest marginal WTP for this group (c.£65), Class-2 shows the lowest WTP (c.£20). The marginal WTP values for innovations targeting ‘drug users’ also show difference across classes: while Class-2 and Class-3 are indifferent in their WTP for innovation targeting ‘drug users’ with respect to the mental health patients, Class-1 is willing to pay less for innovations targeting this group. Another interesting result is the respondents’ valuation of innovations mostly designed for people with obesity. While the smallest class, Class-3 does not value innovations targeting people with obesity significantly different from the one for mental health patients, Class-1 and Class-2, all else being equal, show negative preferences and WTP for these innovations targeting people with obesity. This issue aside, we note that the confidence intervals for all attributes in Class-3 are generally wider than other classes, which would imply that the marginal WTP estimates for Class-3 are less precisely estimated compared to other classes.

The sample also shows variations in marginal WTP for ‘target age’: Class-2 is indifferent in their WTP for innovation targeting adult and young people with respect to elderly, Class-3 is not willing to pay for innovations targeting these two groups. As for the implementation time, people in all classes do not show significant difference in their WTP with respect to the quickest implementation time. The strength of the evidence behind innovations is also valued differently. Relative to those underpinned by scientific studies, respondents in all classes are not willing to pay for innovations that are not scientifically proven. Expert opinion is not valued positively in Class-1, but valued not significantly differently from the scientific studies in Class-2 and Class-3. As for the valuation of potential health gain, higher expected health benefits from an innovation result in higher marginal WTP. While Class-1 and Class-2 are willing to pay not significantly differently for health gains, Class-3 seems to have indifferent preferences for the level of potential health gain with respect to the baseline level.

### Scenario analysis

For ease of interpretation of the results, we explore choice probabilities for different policy options in this scenario analysis. This analysis uses the parameter estimates reported in Table 2 to assess choice predictions under the scale-adjusted latent Class models. For ease of comparison, the choice prediction estimates have been weighted according to the unconditional class membership and scale probabilities. For this analysis, we compare a “no investment” policy option against four different hypothetical policy options presented in Table 4 where we also showed the choice predictions. In this analysis, for simplicity, we assume that these are the only available policy options to the decision-maker. Of course, there are a large number of possible policy options that can be compared.

**Table 4:** **Choice predictions**

	Policy 1	Policy 2	Policy 3	Policy 4
**Target people:**				
People with disability			✓	
People with drug addiction		✓		
People with cancer	✓			
People with mental health problems				✓
People with obesity				
People with asthma				
**Target age:**				
Young (less than 18)		✓		
Adults (18–65)			✓	✓
Elderly (more than 65)	✓			
**Implementation time:**				
Between 6–12 months				✓
More than 12 months	✓			
Between 0–5 months		✓	✓	
**Whether it works:**				
It works but not scientifically proven				
Experts say it works elsewhere in the NHS	✓			
It works and scientific studies confirm this		✓	✓	✓
**Potential health benefits:**				
Moderate health (50%)	✓			
Good health (75%)			✓	
Best health (100%)		✓		✓
**Cost per month:**	£40	£10	£30	£15
**Choice probability**	0.78 (0.69-0.87)	0.43 (0.39-0.48)	0.79 (0.75-0.83)	0.82 (0.80-0.84)

Figures in parentheses are confidence intervals at the 95% level, obtained using the delta method.

The first policy option targets elderly cancer patients, takes more than a year to implement, result in moderate health gain (50%), had expert consultation, and costs as high as £40 per month from individuals’ tax payments^c^. The second policy option targets young people with drug addiction, has quickest implementation time, scientifically proven, results in the best health gain, and costs quarter of the Policy 1 (i.e., £10) per month. Third policy involves investing in an innovation aiming adult population with disability. It has similarities to the Policy 2: scientifically proven and has quickest implementation time. It, however, results in less health gain as compared to the Policy 2 and costs three times more (i.e., £30). The last policy option, the Policy 4, is benefiting adults with mental health problems, takes 6–12 months to take in place in the NHS, confirmed by scientific studies, and results in moderate health gain. This policy requires £15 per month from individuals’ tax contributions.

The results of the scenario analysis reveals that irrespective of the policy option assumptions respondents would prefer the hypothetical innovations to be implemented over the ‘no investment’ options. In particular, respondents are more likely to choose the Policy 4, followed by the Policy 3, Policy 1, and Policy 2. Although ‘cancer’ is being the most prioritised target group, as shown in the results in Table 2, in this hypothetical scenario analysis, innovation targeting cancer patients in Policy 1 is not the most desired option. Similarly, although the Policy 2 results in better health gain, quicker implementation time with less uncertainty around its effects and costs less than other policy options, it is the least preferred policy scenario. On the other hand, Policy 4 that has more health gain, longer implantation time with half the cost of Policy 3 is the most likely to be chosen. As illustrated in this analysis, weights given to the importance of policy features and how respondents make trade-offs between them affect their acceptability.

## Discussion

Prioritising scarce resources for investment in innovation in publically funded health systems is unavoidable. Many healthcare systems wish to foster transparency and accountability in the decisions they make by incorporating the public in decision-making processes. This paper presents a unique conceptual approach exploring the public’s preferences for health service innovations. It involves viewing healthcare innovations as ‘bundles’ of characteristics, rather than dealing with specific innovations in isolation, such as new ways of ‘Chlamydia screening’ or developing new ‘orthopaedic services’. This decompositional view of innovations, as the ‘sum-of-their-parts’ , allows policy-makers to compare numerous competing health service innovations without repeatedly administering surveys for each specific innovation choice.

Methodologically, the research shows how the use of DCE technique, when combined with scale-adjusted LC models explains heterogeneity in public preferences and willingness to pay for health service innovations. To our knowledge, this is the first study to quantify the public’ preferences for health service innovations, described broadly, using a DCE technique with a scale-adjusted LC analysis approach.

The estimation results revealed heterogeneity in respondents’ preferences and WTP for health service innovations. We identified three consumer classes who could be described as ‘concerned’ about spending tax income on certain innovations. Each latent class is also composed of two consumer subgroups having different scale (error variance). This is also referred to as “certainty” [52–54]. According to the results, we found that 35% of the respondents have a higher level of certainty in their choices.

Although there were important differences in preferences for health service innovation investment choices, people in each class, in general, preferred the funds to be spent on innovations that are scientifically proven, have potential health benefits, take less time to implement, and cost less. The research, however, highlighted the contentious nature of policy decision criteria such as ‘age’ [11, 58, 59] and ‘targeting particular populations’ [11, 34, 60]. In the UK, NICEdoes not promote age as a *de facto* basis for priority setting [61]. However, some researchers (e.g., Roberts *et al.* [62]) have described ‘age discrimination’ in local services. Our findings are mixed, as it is found in the literature. While the majority of the respondents preferred innovations targeting ‘young’ or ‘adults’ , a minority (12%) preferred targeting the ‘elderly’. Given we observed no significant differences between classes in terms of respondents’ ages or gender, this finding is interesting. A number of empirical studies also found a lower priority afforded to older people [34, 63–65]. Kappel and Sondøe [33] argue that, other things being equal, young people should be prioritised in distributing limited resources; “either because resources will generally be more useful when given to young people, or because they have lived less life and therefore ‘deserve’ the health improvement [11], p.201]” - a so-called ‘fair innings’ argument. Lewis and Charny [66], found a similar picture. They asked respondents to choose between two people alike in all respects other than age, for a treatment. They found that the respondents had a very strong preference for 5 year olds over 70 year olds, and a strong preference for 35 years old over 60 years old. A qualitative analysis by Schwappach et al. also found “moderate evidence that the public tends to favour the young over the elderly in health-care allocation, although the existence and strength of these preferences varies across countries, study designs and context of questions” [59], p.212].

Conversely, other studies have found significant support for the idea of treating people of all ages equally [67–69], as we found in the latent Class-2 for adult and young people, which was 34% of our sample. Anand and Wailo [67] asked 144 people in Leicestershire, UK to consider how limited NHS funds should be allocated between two types of patients differing only in age. They first asked respondents to choose between groups where age differences were large (80 versus 40), and then gradually decreased the differential until groups differed by one year (41 versus 40). Respondents rejected the use age as a rationing device, even with large differences between age groups and, therefore, large differences in potential health life years saved (or lost).

Different health issues differentially impact on specific populations. Our sample provides evidence of different desires to invest in innovations targeting specific groups, such as people with cancer, disability, drug addiction, or obesity. Three distinct views emerged but in general respondents preferred spending on people with cancer and did not prefer pending on ‘unpopular’ groups, such as people with drug addiction and obesity. In qualitative responses in the survey some individuals took the stance that drug addiction or obesity was self-induced and therefore should be given lower priority. Others have found similar results [34, 42, 60, 66]. Crisp et al. [70], in a UK-based survey, found that their sample viewed drug addiction and alcoholism most negatively and frequently believed people with drug addiction or alcoholism be responsible for their disorders. A telephone survey study by Schomerus et al. conducted with 1012 people in Germany showed that, “perceived personal responsibility exerted significant influence on resource allocation decisions: the less personally responsible sufferers were regarded to be for their alcoholism, the more likely resources for alcoholism were chosen not to be cut [71], p.208]”.

Our study provides policy-makers with the public’s valuation of (and acceptability of) various healthcare innovation scenarios. Importantly, we do this by trading off clear and explicit criteria rather than opaque criteria or procedures [72–74]. The marginal utilities allow us to calculate the overall value attached to competing innovation options and choose the one that the public values the most. Our scenario analysis presented comparisons of a number of hypothetical policy options that involve investment in health service innovations.

Commensurate with its exploratory stage of development, our study has some limitations. Notwithstanding the use of pilot surveys and focus group discussions our questionnaire was limited by the range of characteristics used. We did not for example look at ‘sustainability’ or ‘ease of use of potential innovations’. In defence, we present three main reasons for limiting the characteristics used. First, when a large number of attributes is used in a choice experiment, individuals may tend to use heuristics or lexicographic decision rules, rather than trading-off characteristics [23]. Second, when the number of attributes increases in DCE, the number of choice sets that need to be presented to respondents also increases. Too many choices may impose too high a cognitive burden for respondents. Third, the focus group discussions and interviews with managers from an NHS Foundation Trust helped us in the final selection of attributes that are deemed to be realistic, applicable to a wider range of innovation options, and consistent with decision criteria used at NHS. Future studies, however, should take into account the characteristics that were not included in the study to give a fuller picture. The other limitation is that whilst the sample was reasonably representative of the *general* population (West Yorkshire), the average age was slightly higher than the regional average. Future research will focus on recruiting younger people who are less well represented in this study. Finally, we acknowledge that our relatively small sample prohibits the generalising from our findings to the wider population, and thus requires careful attention in the interpretation of the results. Future research should consider looking at these issues while increasing the sample size.

## Conclusions

Discrete choice approaches can help policy makers, those designing innovations, and decision-makers in health services make more of the public’s views on how services invest their money. This study presents an application of DCE and scale-adjusted latent class modelling that explores the general public’s preferences for health service innovations and how these preferences vary. The methods and findings shed light onto the ways public preferences can be used by policy-makers to support their decision-making and ultimately help make the process of deciding ‘who gets what’ more visible and open to challenge.

### Consent

Return of the completed questionnaires from the general public was taken as implying consent for the publication of this report and any accompanying images.

## Endnotes

^a^The focus group was formed by invitation and involved one-time interview that lasted about two hours. Overall fifteen people were invited via email to participate in the study. Our aim in this purposive sampling was not to recruit a representative sample, but to identify inductively a list of innovation characteristics before interviewing members of the public in a conventionally generalizable, probabilistic, way using conjoint surveys.

^b^Our initial pilot involved 60 people from a range of demographic categories and occupations at the University of York. The pilot yielded data that could be used to identify confusions, lack of understanding, and the time needed to complete the survey. After this first pilot, we formally piloted the survey with 50 people from the general public, NHS managers, and patient panel members of the Bradford Institute for Health Research and NHS Foundation Trust to revise some of the descriptions and modes of presentation used in the questionnaires.

^c^We note that asking a monthly fee may artificially inflate the WTP values, as compared to asking either a one-off or annual sums [75].

^d^The response rate was 20%, which is higher than the survey rate of NHS Foundation Trust that was conducted in the region for another health context looking for the general public’s views about health services.

During administration of questionnaires we did not have any major issues. We excluded 18 questionnaires (3%) from the analysis as they were either completely empty or only responded to a few demographic questions.

^e^When block designs used, it is possible to observe one or more of the latent classes to be driven by a particular block. In order to see if this is the case in this particular study, we included ‘blocks’ as a predictor in the Latent Class model. As a result of the analysis, we did not observe any statistically significant ‘blocks’ effect, and thus conclude that the latent classes are not driven by a particular experimental design block, but by preferences.
